# Recovery from antibody-mediated biliary ductopenia and multiorgan inflammation after COVID-19 vaccination

**DOI:** 10.1038/s41541-024-00861-9

**Published:** 2024-04-08

**Authors:** Alan Chang, Yung-Ming Jeng, Cheng-Maw Ho, Po-Huang Lee

**Affiliations:** 1 Department of Medical Education, National Taiwan University Hospital and College of Medicine, Taipei, Taiwan; 2 Department of Surgery, National Taiwan University Hospital and College of Medicine, 7 Chung-Shan South Road, Taipei, 100 Taiwan; 3 National Taiwan University Hospital, Department of Pathology and College of Medicine, Taipei, Taiwan; 4 Hepatitis Research Center, National Taiwan University Hospital and College of Medicine, Taipei, Taiwan; 5 Department of Surgery, E-Da Hospital, I-Shou University, Kaohsiung, Taiwan

**Keywords:** RNA vaccines, Autoimmune hepatitis

## Abstract

The coronavirus disease 2019 (COVID-19) pandemic has caused significant morbidity and mortality. Spike messenger RNA (mRNA)–based vaccines against severe acute respiratory syndrome coronavirus 2 may contribute to immune-mediated injuries. Here we present a case of a previously healthy 47-year-old man, who developed progressive jaundice 2 weeks after receiving his 3^rd^ COVID-19 vaccination (1^st^ mRNA-based vaccine). Apart from elevated serum total bilirubin levels (peaked at >70 mg/dL), deteriorating renal (blood urea nitrogen: peak, 108.5 mg/dL; creatinine: peak, 6 mg/dL) and exocrine pancreas (amylase: peak, 1717 U/L; lipase: peak, 5784 U/L) profiles were also seen. Vanishing bile duct syndrome characterized by ductopenia and cholangiocyte vacuolation, positive C4d deposition, and high titer of anti-angiotensin II type 1 receptor antibody consistently explain the overall antibody-mediated pathogenesis resembling antibody-mediated “rejection” in the solid organ transplant setting. Corticosteroids and plasmapheresis were administered, leading to gradual resolution of the symptoms, and the jaundice completely resolved 2 months later. In conclusion, we reported a case of antibody-mediated multiorgan injury after an mRNA COVID-19 vaccine, characterized by severe cholangiopathy. The patient recovered with corticosteroids and plasmapheresis, and long-term follow-up is necessary.

## Introduction

The coronavirus disease 2019 (COVID-19) pandemic, as reported by the World Health Organization, has led to over 762 million confirmed cases and 6 million deaths^1^. Given the pandemic’s rapid spread and severe complications, several vaccines have been authorized for emergency use, including adenovirus vector-based vaccines (Jassen), messenger RNA-based vaccines (BNT162b2 mRNA COVID-19 vaccine (Pfizer/BioNTech), and mRNA-1273 sudden acute respiratory syndrome coronavirus 2 (SARS-CoV-2) vaccine (Moderna). While these mRNA-based vaccines have demonstrated good safety and efficacy overall^2–4^, they have been used worldwide for the first time in human history, and rare adverse events have been reported, such as myocarditis^5^, rhabdomyolysis^6^, vaccine-induced immune thrombotic thrombocytopenia^7,8^, and autoimmune diseases^9,10^. While cases of autoimmune hepatitis^11–20^ and kidney diseases^21–24^ have been reported individually, there is limited information regarding multiorgan injuries post-vaccination^25,26^, and the underlying mechanisms remain poorly understood. Here we present a case involving severe cholangiopathy and multiorgan injury (liver, kidney, and pancreas) following COVID vaccination and explore the potential underlying mechanisms.

## Results

### Initial presentation

A 47-year-old Asian man with no history of smoking or alcohol consumption was referred due to progressive jaundice potentially necessitating liver transplantation. His symptoms initially appeared as yellowish skin and generalized itchiness in early May 2022. His medical history was unremarkable except for recent vaccinations. He had received three COVID-19 vaccines: the ChAdOx1 nCoV-19 (AstraZeneca) on July 26, 2021 and December 29, 2021, and mRNA-1273 SARS-CoV-2 (Moderna) on April 26, 2022. Notably, he observed tea-colored urine on May 10, 2022, 2 weeks after the third vaccination. No other symptoms of discomfort were reported. He sought medical attention at a local hospital, where tests revealed elevated serum total bilirubin levels (3.9 mg/dL on May 20, increasing to 32 mg/dL on May 27), abnormal liver enzyme levels (aspartate transaminase [AST] at 234 U/L; alanine transaminase [ALT] at 542 U/L on May 20), and declining renal function (creatinine [Cre] at 2.81 mg/dL on June 13). During subsequent follow-up visits, his total bilirubin and creatinine levels rose to 55 mg/dL and 3.93 mg/dL, respectively, on July 4. Due to this progressive deterioration, he was admitted to the hospital on July 4, 2022. Extensive evaluations ruled out any history of prior hepatitis A, B, or C infections, and the patient had no risk factors for autoimmune diseases, such as a positive family history, current medication use, or recent infections. Abdominal computed tomography (CT) displayed no signs of cholelithiasis, biliary tract dilatation, cirrhosis, or portal hypertension. On July 8, 2022 (day 0), he was referred to our transplant center.

**Fig. 1:**
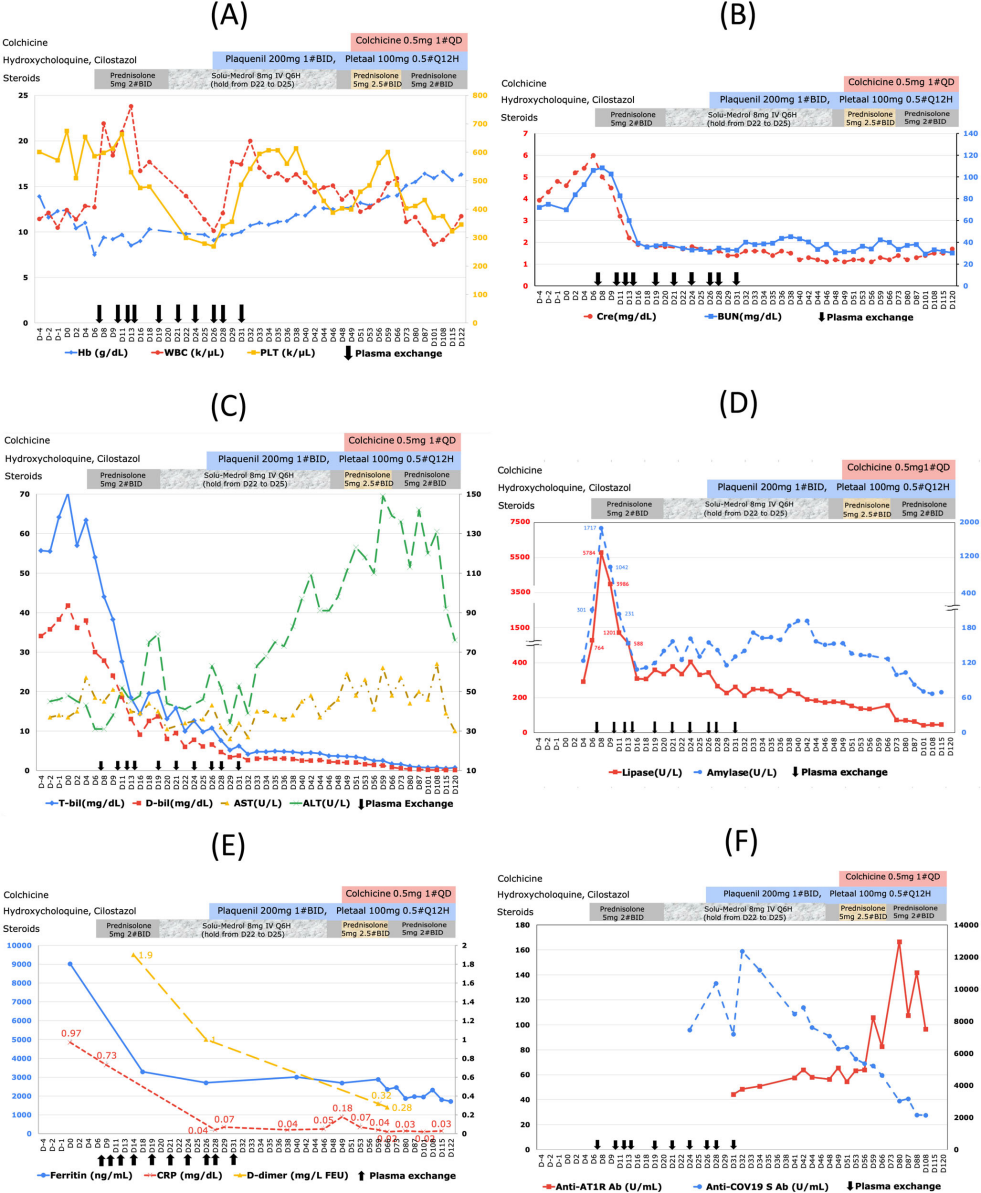
Laboratory trends in a case of post-COVID-19 vaccination severe jaundice and multiorgan inflammation. Hemogram (**A**), kidney (**B**), liver (**C**), pancreas (**D**), inflammation (**E**), and antibodies (anti-SARS-CoV-2 Spike antibody & angiotensin II receptor type 1 (AT1R) antibody) (**F**). Day 0 is the date of admission and doses and duration of medications are at top. Improvement was noted after starting steroids and plasma exchange (D7). Hb drop was noted after liver biopsy (D5). Reference of positivity: anti-SARS-CoV-2 Spike antibody (≥0.8 U/mL, quantitative Roche Elecsys® Anti-SARS-CoV-2 S assay), angiotensin II receptor type 1 (AT1R) antibody (>17 U/mL, enzyme immuno-assay (EIA), CellTrend GmbH). Normal ranges of laboratory testes other than antibodies were presented in Supplementary Table S1. ALT alanine transaminase, AST aspartate transaminase, BUN blood urea nitrogen, Cre creatinine, CRP C-reactive protein, D-bil direct bilirubin, Hb hemoglobin, PLT platelet, T-bil total bilirubin, WBC white blood cell.

Upon admission, the patient exhibited yellowish skin and sclera, but he did not have clay-colored stools. Laboratory analyses revealed cholestatic liver injury with the following results: total bilirubin of 64.2 mg/dL, ALT of 46 U/L, AST of 38 U/L, alkaline phosphatase (ALP) of 361 U/L, international normalized ratio of 0.95, and Cre of 4.8 mg/dL. The fractional excretion of urea was 54.9%, which suggested intrinsic renal disease. The patient had adequate urine output of approximately 3000 mL/day during his hospital stay. An abdominal ultrasound performed on day 4 showed no cholelithiasis, dilatation of the common bile duct, normal pancreatic parenchyma, and bilaterally enlarged kidneys (15 cm in length, reference range: 9–12 cm). Hemogram results in the early clinical course indicated leukocytosis and thrombocytosis (Fig. 1A). Laboratory data on day 6 revealed worsening kidney function, with a Cre level of 6 mg/dL and BUN level of 106 mg/dL (Fig. 1B). Infective surveys were negative for SARS-CoV-2 RNA PCR and nucleocapsid antibody, hepatitis E immunoglobulin M (IgM), anti-HIV antibody, and leptospiral antibody–IgM. Previous but not recent exposure to cytomegalovirus, Epstein-Barr virus, and herpes simplex virus-1 was noted. Autoimmune surveys conducted via immunofluorescence assay were positive for anti-smooth muscle antibody (1:40) but negative for antinuclear antibody, anti-neutrophil cytoplasmic antibody, anti-mitochondrial antibody, and anti-liver–kidney microsomal antibody. The patient’s immunoglobulin G levels were within the normal range. An inflammatory profile revealed significantly elevated ferritin level (9015.9 ng/mL; normal range: 24–336 ng/mL), slightly elevated C-reactive protein (CRP) levels (0.97 mg/dL), and an increased erythrocyte sedimentation rate of 34 mm/h (Fig. 1E). Transferrin and ceruloplasmin levels were within normal ranges. Specific antibodies, including anti-SARS-CoV-2 spike antibody and AT1R antibodies, were utilized for further mechanistic investigation (details below) (Fig. 1F).

**Fig. 2:**
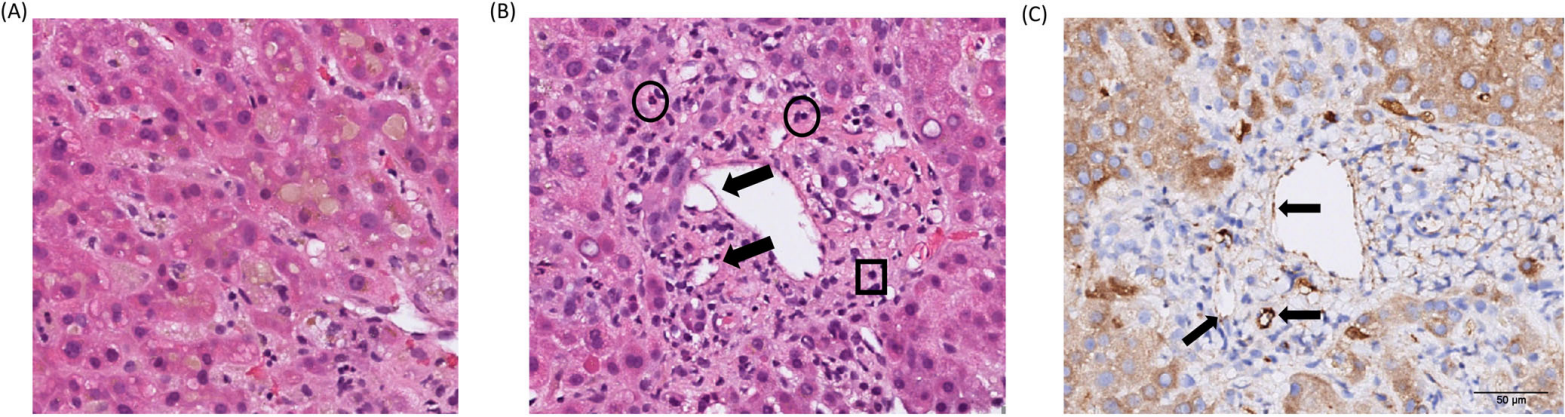
Liver biopsy in a case of post-COVID-19 vaccination severe jaundice and multiorgan inflammation. Histopathological (**A**, **B**) and immunohistopathological (**C**) examinations of liver biopsy. **A** Marked centrilobular cholestasis. **B** Mild lymphocytic (square) and neutrophilic (circle) infiltration in the portal areas with bile duct damage and cholangiocyte vacuolation (black arrows). **C** Positive C4d deposition in portal venous and capillary endothelial cells (black arrows). 200X.

Percutaneous liver biopsies conducted on day 5 after admission revealed centrilobular cholestasis and bile duct damage, characterized by cholangiocyte vacuolation. The bile ducts were lost in some of the portal areas (Fig. 2A). These findings were consistent with obstructive cholestasis resulting from the inflammatory destruction of the intrahepatic bile ducts, a condition known as vanishing bile duct syndrome (Fig. 2A). Importantly, this pattern did not suggest an infectious etiology. Additional immunostaining unveiled positive C4d deposition in endothelial cells, as demonstrated in Fig. 2B. This finding strongly indicated antibody-mediated liver injury, resembling the pattern of antibody-mediated rejection (AMR) seen in solid organ transplantation.

Initial management included supportive medications for symptom relief and short-term empirical antibiotics (doxycycline and levofloxacin, ended immediately after liver biopsy) for suspected atypical infection with multiple organ involvement in inflammation (liver, kidney, and pancreas). We initiated oral prednisolone at a dosage of 10 mg twice daily on day 6, immediately following the receipt of the biopsy results.

On day 7, we initiated plasma exchange as a bridge to liver transplantation. The patient underwent two courses of plasma exchange, with the first course (four times) performed on days 7, 10, 12, and 14. The laboratory data on day 8 after the first plasma exchange showed a decrease in serum bilirubin levels and an improvement in renal function (as indicated in Fig. 1B, C). The serum bilirubin levels showed a declining trend following a series of plasma exchange (as shown in Fig. 1C), dropping from 18.45 mg/dL on day 13 to 14.35 mg/dL on day 16. However, they later increased to 19.49 mg/dL on day 18 when plasma exchange was not continued. A similar pattern was observed for the direct bilirubin levels. Therefore, we performed additional six times of plasma exchange and switched the steroids from oral prednisolone back to intravenous methylprednisolone 8 mg every 6 h since day 20. Intractable pruritus, especially at night and unresponsive to antihistamines and cholestyramine, seemed subjectively responsive to an increased dose of steroids. We tapered off the steroids from day 22 to day 25 and observed a transient increase in serum bilirubin levels, along with increased levels of amylase, lipase, and a decline in renal function. On days 22 and 42, serum bile acid levels were 122 µM and 40 µM, respectively. The normal range is <10 µM, while levels exceeding 50 µM are considered elevated.

On day 6, the patient experienced a transient episode of mild epigastric pain. Subsequently, amylase and lipase levels reached their peak at 1717 U/L and 5784 U/L on day 8, respectively (compared to initial data 123 U/L and 290 U/L on day 4) (see Fig. 1D). Abdominal CT on day 5 and follow-up ultrasound scans on day 8 and 19 revealed the absence of gall stones, common bile duct stones or dilatation, and the pancreas parenchyma remained intact without necrosis or swelling. Under the impression of mild acute pancreatitis, temporary parenteral nutritional support was initiated, and oral food intake was not resumed until amylase levels dropped to 108 U/L and lipase levels to 307 U/L on day 16.

### Intervention, recovery, and mechanistic investigation

Recent literature suggests that immune-mediated liver injury following COVID-19 vaccination and the potential mechanisms and antibody candidates involved warrant further investigation^19,20,27^. In this case, we observed a decreasing trend in the anti-SARS-CoV-2 spike antibody level (peak, 12,371 BAU/mL on day 32; positive, ≥0.8 BAU/mL), the vaccine-induced antibodies against the viral spike protein, measured using a quantitative Roche Elecsys® Anti-SARS-CoV-2 S assay (Fig. 1F). This decline in antibody levels could possibly be attributed to the effects of therapeutic plasma exchange (major) and a gradual ‘natural’ fading over time post-vaccination (minor)^28,29^. The peak level was considered high compared to a COVID-19-naïve population receiving a similar course of COVID-19 vaccination with a geometric mean concentration of anti-SARS-CoV-2 spike IgG level of 3898 BAU/mL (range: 3303–4600 BAU/mL, 28 days post boost dose), indicating a stronger immune response^28^. The clinical course in this case suggested adverse events associated with COVID-19 vaccination, supported by clinical findings and chronological correlation. COVID-19 mRNA vaccines are known to potentially induce systemic inflammation and multiorgan injury by provoking proinflammatory responses and eliciting autoimmune reactions^9,26,27^. Three main hypothetical mechanisms through which COVID-19 vaccines could trigger autoimmunity are: molecular mimicry (immune cross-reactivity due to similarities between certain vaccine components and specific human proteins), the production of autoantibodies (e.g., anti-platelet factor 4 antibodies), and the specific vaccine adjuvants (by triggering innate inflammatory responses)^9,30^. The biopsy results and the clinical manifestations in this case, along with the insights derived from the non-HLA antibody-mediated graft injury, particularly the presence of anti-angiotensin II type 1 receptor (AT1R) antibodies, prompted us to further investigate the potential involvement of these antibodies in the pathogenesis. Ideally, the vaccine simulates an actual infection by mimicking the virus and the immune system reacts similarly to an actual infection. However, increased AT1R antibody levels have been observed in cases of multiorgan tissue damage and acute respiratory distress syndrome in COVID-19 patients^31,32^. We found that the AT1R antibody level in this patient was elevated (peak, 166.61 U/mL; positive, >17 U/mL) (Fig. 1F). AT1R antibody levels were determined in the serum using a capillary electrophoresis–marked enzyme immunoassay developed at CellTrend GmbH according to the manufacturer’s instructions.

Rheumatologists recommend pharmacological combinations of hydroxychloroquine and cilostazol to treat thrombotic antibody-mediated inflammation. Although ferritin levels decreased during the clinical course, they remained highly elevated compared to the upper limit of normal (Fig. 1E). The CRP level exhibited a similar trend but returned to normal after day 28 (Fig. 1E).

As jaundice gradually improved, steroids were tapered from intravenous methylprednisolone to oral prednisolone at a dose of 10 mg twice daily starting on day 47. Additionally, colchicine was introduced, and the oral prednisolone dose was titrated to 12.5 mg twice daily in response to fluctuations in liver enzyme levels (Fig. 1C), even as serum bilirubin levels continued to decrease (peak AST, 64 U/L on day 113; ALT, 149 U/L on day 64). The patient was discharged on day 57 and continued with regular outpatient visits. Throughout the course of treatment, the patient’s mental status was within normal limit, and he was able to ambulate and articulate freely. Details of the follow-up laboratory results and medication usage are presented in Fig. 1. On day 171, abdominal magnetic resonance imaging and cholangiopancreatography revealed unremarkable findings in the liver, gall bladder, kidneys, and pancreas.

## Discussion

To our knowledge, the involvement of the liver, kidney, and pancreas in this case represents the first instance of vanishing bile duct syndrome with multi-organ involvement associated with the COVID-19 vaccine and correlated with elevated anti-AT1R antibodies. Data from the Vaccine Adverse Event Reporting System suggest that abdominal pain and diarrhea are the most common gastrointestinal side effects after COVID-19 vaccination, and 238 incidences and 79 deaths have occurred from liver failure post-vaccination in the USA^33,34^. Recent case reports have indicated that the mRNA vaccines produced by Pfizer-BioNTech and Moderna are most commonly associated with autoimmune hepatitis^12–15^. While transaminasemia has been reported rarely after COVID-19 vaccination, it may explain the initial increase in transaminase levels observed in our patient^35,36^. The mean time from vaccination to symptom onset was 13 days (range, 4–26 days)^30^, with most cases of vaccine-induced hepatitis exhibiting hepatocellular injury^11–18,37–39^. However, in this patient, cholestatic injury was the predominant feature. Few cases, reported by Mann et al. and Zafar et al., involved patients with no history of autoimmune disease or COVID-19 infection who developed cholangiopathy after COVID-19 vaccination, which resolved a few days under conservative treatment^20,35^. In contrast to the liver biopsy findings in one of the aforementioned cases, which showed features compatible with drug-induced cholangiopathy, characterized by portal inflammation and ductitis, the biopsy in our case revealed a different pattern consistent with vanishing bile duct syndrome. This pattern included marked centrilobular cholestasis, bile duct damage with cholangiocyte vacuolation, and a scant eosinophilic infiltrate. In an international cohort of 59 individuals with acute hepatitis arising after SARS-CoV-2 vaccination, liver histology showed lobular hepatitis in 45 patients (76%) and portal hepatitis in 10 patients (17%). Autoimmune serology revealed anti-antinuclear (74%, negative in our case) and anti-smooth muscle antibodies (61%, positive in our case)^40^. Corticosteroid therapy may be beneficial in patients with immune-mediated features or severe hepatitis^11,14^. Multiorgan dysfunction after COVID-19 mRNA vaccination has been reported in healthy individuals^25,26^. It’s challenging to directly link the mRNA-1273 COVID-19 vaccine to the observed syndrome. However, considering this patient’s prior health status, the absence of other potential causes, and the timing, a plausible connection can be inferred. Ni et al. reported that the overall angiotensin-converting enzyme 2 (ACE2)–binding mode of the SARS-CoV-2 receptor-binding domain is nearly identical to that of the SARS-CoV receptor-binding domain, which also uses ACE2 as the cell receptor^41^. The broad expression of ACE2 in various cell types and tissues results in an expanding tropism of SARS-CoV-2 for multiple critical organs, such as heart, pancreas, and kidneys^42,43^. Consequently, SARS-CoV-2 infection can lead to ACE2 signaling downregulation and dysregulation of the renin-angiotensin system (RAS)^42,43^. For mRNA-based vaccines (e.g., mRNA-1273 SARS-CoV-2 vaccine [Moderna] and BNT162b2 mRNA Covid-19 vaccine [Pfizer/BioNTech]), potential biological mechanisms for an increased risk of immune-mediated diseases include mRNA’s intrinsic immunostimulatory properties. The vaccine’s end-protein (spike protein), by mimicking the behavior of the virus, can bind to ACE2, disrupt the body’s normal RAS, and cause endothelial injury^9,27,44,45^. Endothelial damage can trigger the development of anti-endothelial antibodies (e.g., anti-AT1R antibody) which can attack multiple organs^46^. If there is a dysregulated, prolonged innate immune response, these changes may cause systemic inflammation and contribute to the development of autoimmune reactions and multiorgan injuries in some individuals after COVID-19 vaccination^9,27,44,45^. The role of anti-AT1R antibody was investigated in our case based on the resembling features of its involvement in both COVID-19-related multiorgan injury and AMR in solid organ transplantation. The diagnosis of chronic active AMR in transplantation requires morphological evidence of chronic tissue injury plus two of three key findings: the presence of donor-specific antibodies, positive C4d staining, and compatible tissue damage^47^. This patient exhibited a pathological picture of vanishing bile duct syndrome, C4d deposition in endothelial cells, and a highly elevated level of anti-AT1R antibody, which mirrors the features of non-HLA AMR in solid organ transplantation^48–50^. AT1R is broadly expressed in various cells, tissues, and organs, including vascular endothelial cells, smooth muscle cells, inflammatory cells, and the brain, lungs, heart, liver, adrenal cortex, kidneys. Therefore, anti-AT1R antibodies can damage endothelial and vascular smooth muscle cells, leading to elevated levels of transcription factors associated with pro-inflammatory responses in multiple organs^51^. Besides, while most vaccinated individuals with anti-SARS-CoV-2 spike antibodies do not report side effects, this case had higher-than-average anti-SARS-CoV-2 spike antibody levels, which might indicate a stronger immune response and dysregulated inflammation, potentially leading to RAS overactivation and organ damage. The notion is supported by the fact that the level of anti-spike antibodies post SARS-CoV-2 exposure is correlated with broad autoantigen recognition and the number of autoantibodies^52^. Overall, in this case, the investigation favored the possibility of anti-AT1R antibodies playing a contributing role in the patient’s condition, potentially in conjunction with a dysregulated RAS. Based on clinical picture mimicking AMR with the presence of antibodies (anti-AT1R), C4d staining, and compatible antibody-mediated tissue injury, we propose that anti-AT1R, which can be seen in AMR of liver and kidney transplant, probably induced after COVID vaccination, was pathogenic in this patient. Further data should be examined to determine if similar complications, such as multiorgan injury, occur with other mRNA or bivalent vaccines. We also call for the need of further investigation to validate the role of spike hypothesis^27^ in the upstream perturbation ahead of injury by anti-AT1R antibody. Of note, the cholemic nephropathy due to extremely high serum bilirubin levels and strong T-cell responses from the mRNA vaccine might also contribute to renal injury in this patient, which cannot be completely ruled out^22–24^.

In conclusion, here we reported a case of severe cholangiopathy and proposed that the mechanism mimicking AMR is the main contributor to COVID vaccine–mediated multiorgan injury. Thus, AT1R is a highly probable candidate. Corticosteroids and plasmapheresis are useful for its resolution; however, long-term follow-up is required.

## Methods

The research was conducted in accordance with both the Declarations of Helsinki and Istanbul. The Institutional Review Board of National Taiwan University Hospital approved this study (202004053RINB). Because this study retrospectively analyzed data through a chart review, the Institutional Review Board of National Taiwan University Hospital waived the need for informed consent.

### Histopathologic assessment

Liver biopsy was performed on day 5 after admission. Paraffin-embedded specimens were subjected to hematoxylin and eosin staining and C4d immunostaining (a polyclonal rabbit anti-human C4d antibody; Cell Marque, Rocklin, CA, USA). The pathologists evaluated the specimens to confirm the diagnosis.

### Laboratory assays

Serum samples were specifically tested for anti-AT1R antibody and anti-SARS-CoV-2 spike antibody.

Anti-AT1R antibody was determined using a CE-marked enzyme immuno-assay (EIA), developed at CellTrend GmbH (https://www.celltrend.de/en/elisa/in-vitro-diagnostika-human/), according to the manufacturer’s instructions. For antibodies against AT1R, a concentration of 10 U/mL was considered the cutoff value (10–17 U/mL borderline and >17 U/mL positive) in line with the manufacturer’s recommendation.

The level of anti-SARS-CoV-2 spike antibody was quantified using the Roche Elecsys® Anti-SARS-CoV-2 S assay. This Roche test is a quantitative (range: 0.4–250 U/mL, whereby the conversion factor of the system’s arbitrary U/mL to BAU/mL is 1.0) total antibody sandwich assay recognizing antibodies directed against the receptor-binding domain (RBD) of the SARS-CoV-2 spike (S) protein. Values exceeding the manufacturer’s specified linear range (250 U/mL) were appropriately diluted as per the manual’s recommendations until the measurements fell within the linear range once more. The final concentration in these instances was determined by applying the dilution factor to the measured units. Samples >0.8 BAU/mL are considered diagnostically positive.

### Reporting summary

Further information on research design is available in the Nature Research Reporting Summary linked to this article.

### Supplementary information

Supplementary Table 1

REPORTING SUMMARY

## Data Availability

The datasets used and analyzed during the current study are available from the corresponding author upon reasonable request.
