# Supplementary material for: Recovery from antibody-mediated biliary ductopenia and multiorgan inflammation after COVID-19 vaccination
Source: NPJ Vaccines. 2024 Apr 8;9:75. doi: 10.1038/s41541-024-00861-9 (PMC11001909; doi:10.1038/s41541-024-00861-9)
Supplement: Supplementary file 1 — Supplementary Table 1 [file 41541_2024_861_MOESM1_ESM.pdf]

Table S1. Normal ranges of laboratory tests in Figure 1

Item	Unit	Normal ranges
Amylase	U/L	29~103
AST	U/L	8~31
ALT	U/L	0~41
BUN	mg/dL	7~25
Cre	mg/dL	0.6~1.3
CRP	mg/dL	<0.3
D-bil	mg/dL	0.03~0.18
D-dimer	mg/L FEU	<0.56
Ferritin	ng/mL	21.8~274.7
Hb	g/dL	13.1~17.2
Lipase	U/L	11~82
PLT	k/μL	150~378
T-bil	mg/dL	0.3~1
WBC	k/μL	3.25~9.16

Abbreviations: ALT, alanine transaminase; AST, aspartate transaminase; BUN, blood urea nitrogen; Cre, creatinine; CRP, C-reactive protein; D-bil, direct bilirubin; Hb, hemoglobin; PLT, platelet; T-bil, total bilirubin; WBC, white blood cell
